# Atypical Growth Pattern of an Intraparenchymal Meningioma

**DOI:** 10.1155/2016/7985402

**Published:** 2016-09-26

**Authors:** Zhen Zeng, Tijiang Zhang, Yihua Zhou, Xiaoxi Chen

**Affiliations:** ^1^Department of Radiology, Affiliated Hospital of Zunyi Medical College, Zunyi, China; ^2^Department of Radiology, Saint Louis University School of Medicine, St. Louis, MO, USA

## Abstract

Meningiomas are the most common primary nonneuroglial extra-axial neoplasms, which commonly present as spherical or oval masses with a dural attachment. Meningiomas without dural attachment are rare and, according to their locations, are classified into 5 varieties, including intraventricular, deep Sylvain fissure, pineal region, intraparenchymal, or subcortical meningiomas. To the best of our knowledge, intraparenchymal meningioma with cerebriform pattern has never been reported. In this paper, we report a 34-year-old Chinese male patient who presented with paroxysmal headaches and progressive loss of vision for 10 months and blindness for 2 weeks. A thorough physical examination revealed loss of bilateral direct and indirect light reflex. No other relevant medical history and neurologic deficits were noted. Computed tomography and magnetic resonance imaging scans showed an irregular mass with a unique cerebriform pattern and extensive peritumoral edema in the parietal-occipital-temporal region of the right cerebral hemisphere. The initial diagnosis was lymphoma. Intraoperatively, the tumor was completely buried in a sulcus in the parietal-occipital-temporal region without connecting to the dura. The histological diagnosis was intracranial meningioma based on pathological examination. Therefore, when an unusual cerebriform growth pattern of a tumor is encountered, an intraparenchymal meningioma should be considered as a differential diagnosis.

## 1. Introduction

Meningiomas are the most common primary extra-axial neoplasms in adults, accounting for up to 30% [1] of all primary intracranial tumors and as high as 35.5% in Asian and African people [2]. Generally speaking, meningiomas have a dural attachment. Meningiomas without dural attachment are rare and are more likely to occur in young males and are most commonly seen in the intraventricular and pineal regions and the Sylvain fissure [3–5]. Intraparenchymal meningioma is extremely rare with only few reported cases [6–8]. To the best of our knowledge, an intraparenchymal meningioma with a cerebriform pattern has never been reported. In this paper, we present a rare case of primary intraparenchymal meningioma with a cerebriform pattern, which was preoperatively misdiagnosed as lymphoma based on conventional CT and MR imaging features.

## 2. Case Presentation

**Figure 1:**
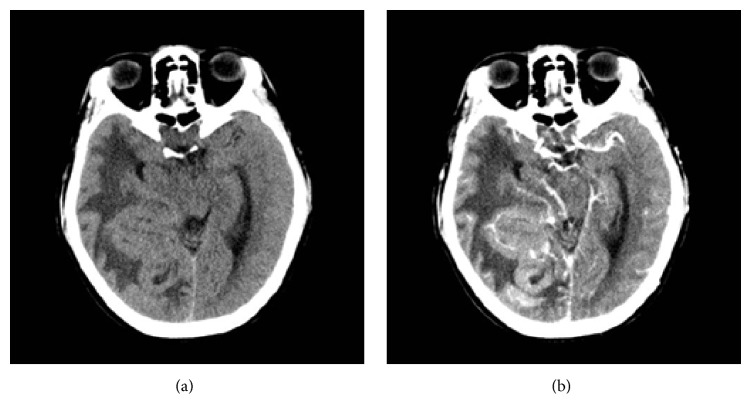
Axial CT images showed a cerebriform and irregular isodense mass with prominent perilesional vasogenic edema (a-b) in the right parietal-occipital-temporal region.

**Figure 2:**
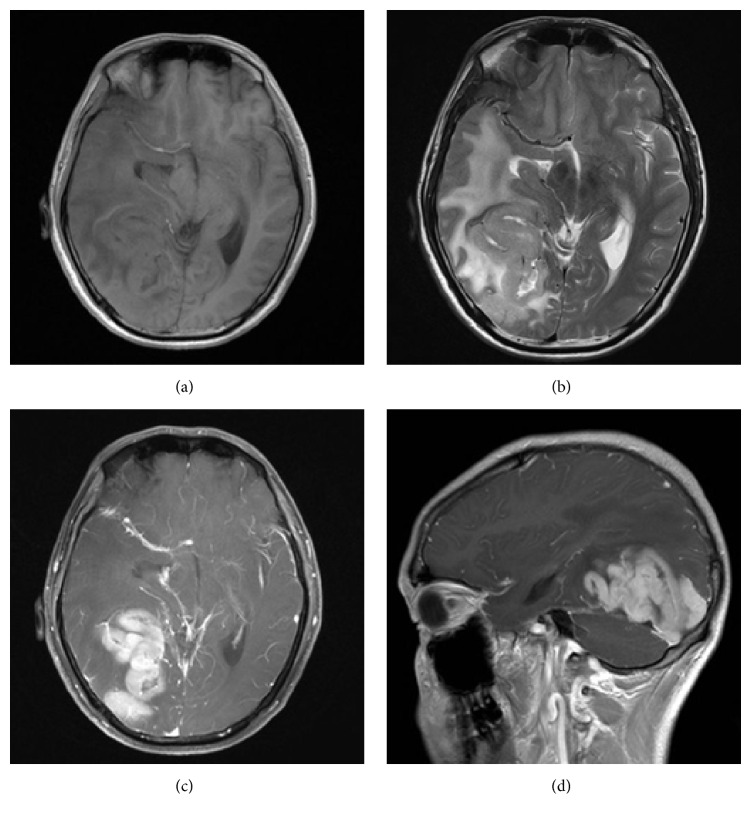
MR images from the same patient as shown in Figure 1. The mass was isointense on T1WI and slightly hyperintense on T2WI. Contrast-enhancement T1WI images showed prominent homogeneous enhancement. The tumor was buried in the cortical sulci without any dural attachment.

**Figure 3:**
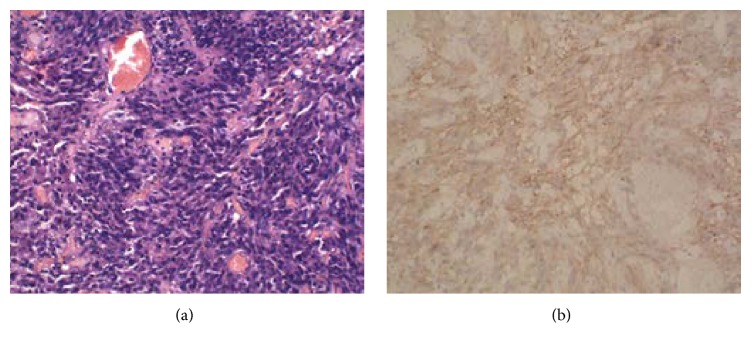
Histopathological manifestations of meningioma with cerebriform pattern. Hematoxylin-eosin staining (×100) revealed a large number of spindle cells rich in* Vascellum*, without distinct karyokinesis (a). Immunohistochemical staining (diaminobenzidine ×20) showed cells positive for epithelial membrane antigen (b).

**Figure 4:**
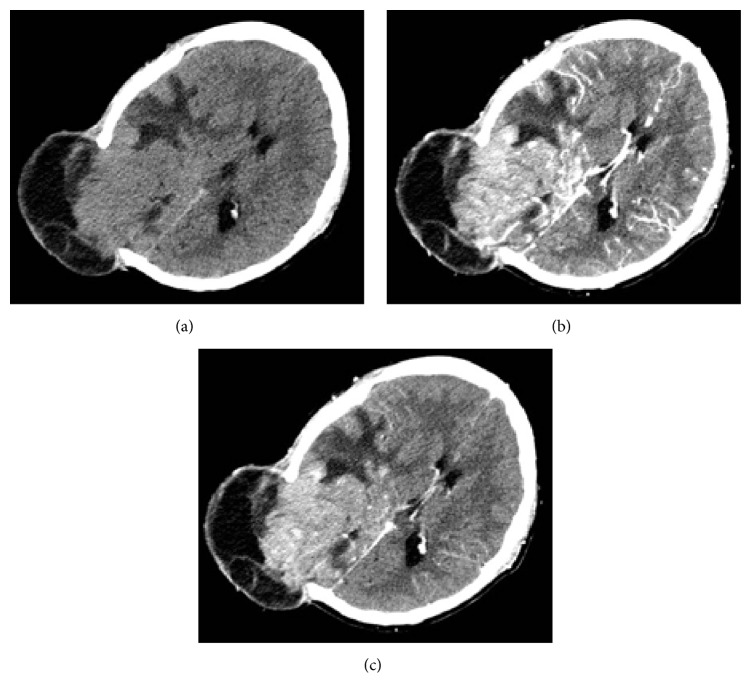
Follow-up CT images showed recurrent tumor herniating through the craniectomy defect. An axial CT image showed an irregular isodense mass with prominent perilesional vasogenic edema located in the right parietal-occipital-temporal lobe (a). Contrast-enhancement CT images showed intense homogeneous enhancement (b, c).

A 34-year-old male patient presented with paroxysmal headaches and progressive vision loss for 10 months and blindness for 2 weeks. Physical examination revealed loss of bilateral direct and indirect light reflexes. No other neurologic deficits and laboratory abnormalities were noted. CT imaging showed an irregular isodense mass with a cerebriform appearance and extensive peritumoral edema in the parietal-occipital-temporal region of the right hemisphere (Figure 1). MR imaging demonstrated a cerebriform, irregular but well-defined mass in the parietal-occipital-temporal region of the right hemisphere. It was isointense on T1-weighted images and slightly hyperintense on T2-weighted images. There was intense homogeneous enhancement after contrast administration. The mass measured 46 cm × 80 cm × 51 cm and demonstrated significant mass effect, resulting in a 13 mm midline shift, compression of brainstem, and effacement of the occipital horn of the right lateral ventricle and ambient cistern (Figure 2). Unfortunately, advanced MRI imaging such as perfusion weighted imaging (PWI) and MR spectroscopy (MRS) was not performed on the initial MRI examination and the patient denied the suggestion of further imaging. Intraoperatively, the tumor was completely buried in a sulcus in the parietal-occipital-temporal region. The tumor was completely resected with careful dissection from the surrounding brain tissues. Macroscopically, the tumor showed rich blood supplies but without a dominant feeding artery. Histopathologically, the tumor showed a large number of spindle cells. There was no distinct karyokinesis. On immunohistochemical staining, the pathology specimen of this tumor was positive for epithelial membrane antigen (Figure 3) but negative for glial fibrillary acidic protein. Ki-67 was 1-2%. Based on the pathological findings, the tumor was diagnosed as a grade II intracranial meningioma according to classification criteria of World Health Organization (WHO). Postoperative radiotherapy was offered to the patient. However, the patient and his family denied the treatment. Twelve months after surgery, the patient returned to the hospital with progressive headaches and a CT examination (Figure 4) revealed recurrence. After surgical resection, the recurrent tumor was determined to be meningioma (WHO grade III).

## 3. Discussion 

In this report, we presented a case of a large intraparenchymal meningioma that likely developed in a long period of time. Despite the significant mass effect caused by its large size and the extensive surrounding edema, no cognitive deficit was detected at presentation, which was likely due to compensation/accommodation to the slow growth of the tumor. However, the patient had lost both direct and indirect pupil light reflexes which may be secondary to optic atrophy resulting in slowly but progressively elevated intracranial pressure.

This case showed an unusual growth pattern of an intraparenchymal meningioma which was characterized by its cerebriform appearance and the involvement of cortical sulci without dural attachment. The etiology of meningiomas without dural attachment is unclear. Some authors presume that such meningiomas arise from arachnoid cells of the pia mater that stretches into the surface of brain or sulcus with perforating blood vessels, while others suggest that the arachnoid cells rest in the brain during the migration progress [3]. The meninges comprise three membranous layers: pia dura, arachnoid membrane, and dura mater. The arachnoidal villi, which are capped by the arachnoidal cells, may stretch into the sulci and the brain parenchyma along with the pia mater. We believe this may explain the cerebriform appearance of the meningioma in our case.

To the best of our knowledge, only 31 cases of intraparenchymal meningiomas have been reported [3–8]. This is the first case of intraparenchymal meningioma with a cerebriform pattern. It has been known that intraparenchymal meningiomas are more likely to occur in young males and can be located in all regions of the supratentorial brain. The most common presenting symptom is seizure followed by headache. Most intraparenchymal meningiomas are of fibrous type, whereas the extra-axial meningiomas are of the meningothelial type. The most common location is the frontal lobe followed by the parietal lobe. The imaging findings of intraparenchymal meningioma are nonspecific and most cases are misdiagnosed preoperatively.

Due to the absence of typical features on CT and MRI scans, the preoperative diagnosis of this disease is extremely difficult. Our case showed several findings leading to the diagnosis of lymphoma: the periventricular location, an isodense or slightly hyperdense mass on CT scans, and isointensity with intense enhancement on T1WI and T2WI. It has been reported that intraparenchymal meningioma may be misdiagnosed as glioma [3], metastatic tumor [6], and cavernoma [4]. Our case and others reported in the literature indicate that conventional imaging technology may not always be reliable in distinguishing intraparenchymal meningiomas from other intra-axial tumors. Advanced MRI techniques are important supplemental means in improving the reliability of preoperative diagnosis and differential diagnosis.

Given their vastly different treatments and prognosis, intraparenchymal cerebriform meningiomas should be differentiated from lymphoma and glioblastoma multiforme (GBM). Brain lymphoma accounts for 1–5% of all brain tumors [9]. It occurs in the brain parenchyma nearly exclusively. Lymphomas have a predilection for the basal ganglia and periventricular and superficial regions. They appear to be isodense or hyperdense on CT and isointense on T1WI and T2WI images with intense enhancement but relatively mild surrounding edema. In immune compromised patients, CNS lymphomas usually have cystic changes and central necrosis. They may show the “clench fist sign” and “cusp angle sign” on contrast-enhancement images. CNS lymphomas typically demonstrate diffusion restriction on diffusion weighted imaging and hypoperfusion on MR PWI. An abnormal lipid peak and increased choline/creatine ratios can be seen on MRS [10]. As CNS lymphoma may be cured by chemotherapy and/or radiotherapy for its sensitive response to chemotherapeutic agent or radiation, when it is difficult to differentiate an intraparenchymal meningioma from a CNS lymphoma, stereotactic biopsy of lesion should be considered to obtain tissue diagnosis before proceeding to gross total resection of the tumor. GBM is the most common primary malignant brain tumor in adults. It usually occurs in the supratentorial white mater, particularly in the frontal and temporal lobes. Imaging characteristics of GBM include invasion of the corpus callosum and involvement of the contralateral hemisphere, producing the so-called “butterfly sign.” Necrosis and cystic changes are common in GBM. Therefore, contrast-enhanced images often demonstrate prominent irregular enhancement in the periphery of the tumor. The aggressive growth pattern of GBM is commonly accompanied by marginal tumor infiltration and extensive surrounding edema. The relative cerebral blood volume in meningioma is remarkably higher than that in GBM on PWI. In addition, meningiomas typically display the type III curve (postenhancement part is below the baseline) on signal intensity-time curve analysis, whereas GBM shows the type II curve (postenhancement part is above the baseline). On MRS, Alanine is commonly observed in meningioma. Based on these imaging features, the cerebriform meningioma may be differentiated from GBM and lymphoma.

In summary, in this paper, we present the cerebriform imaging characteristics of a rare intraparenchymal meningioma and review the imaging findings that could help us differentiate atypical meningiomas from other types of brain tumors such as CNS lymphoma and GBM. Advanced MRI techniques such as MRS and PWI can effectively improve the preoperative diagnosis of this tumor.
